# Effects of rapid repetition of a vascular occlusion test on near-infrared spectroscopy-derived variables in healthy subjects and in critically ill patients

**DOI:** 10.1186/cc12618

**Published:** 2013-06-19

**Authors:** DO Cortes, F Puflea, K Donadello, D de Backer, J-L Vincent, J Creteur

**Affiliations:** 1 Erasme Hospital, Universite Libre de Bruxelles, Anderlecht, Bruxelles, Belgium

## Introduction

Transient ischemia modifies cellular metabolism and microvascular physiology in order to limit damage from future hypoxic episodes, a phenomenon called preconditioning. Near-infrared spectroscopy (NIRS) is a non-invasive technique that, when coupled to a vascular occlusion test (VOT), provides an indirect measurement of muscle oxygen consumption (VO_2_) and microvascular reactivity. We hypothesized that: rapid repetition of a VOT may alter VOT-induced NIRS-derived variables and these changes could reflect preconditioning; and these alterations would be different in healthy volunteers and critically ill patients.

## Methods

Continuous non-invasive measurements of thenar tissue oxygen saturation (StO_2_) were performed using NIRS technology (InSpectra 650; Hutchinson, USA). VOTs were performed by inflating a cuff to 50 mmHg above the systolic pressure for 3 minutes. In a group of healthy volunteers, the VOT was repeated after 5 minutes on day 1, after 15 minutes on day 2 and after 30 minutes on day 3. In a group of critically ill patients, the VOT was repeated after 5 minutes. For each VOT, we calculated the StO_2 _desaturation slope (DescSlope), StO_2 _resaturation slope (AscSlope) and the NIRS VO_2 _as the DescSlope×mean total hemoglobin index over the occlusion time. All statistical analyses were performed using SPSS 19.0 (IBM, USA).

## Results

Twenty-one healthy volunteers (age 29 ± 6 years, heart rate 71 ± 6 bpm, mean arterial pressure 82 ± 6 mmHg) and 18 critically ill patients (age 59 ± 14 years, APACHE II score 21 ± 9, norepinephrine use in 10/18, ICU mortality 22%) were included. In the healthy volunteers, repetition of the VOT was associated with a decrease in the DescSlope and in NIRS VO_2_. This effect was not observed in the critically ill patients (Tables 1 and 2).

**Table 1:** Effects of a repeat VOT on VOT-induced NIRS-derived variables in healthy volunteers

Interval	Variable	First	Second	*P *value
5 minutes	AscSlope	4.2 (3.4 to 4.9)	4 (3.3 to 5)	0.298
	DescSlope	12 (9.2 to 14.5)	9.8 (8.3 to 10.8)	>0.001
	NIRS VO_2_	151 (132 to 171)	131 (118 to 146)	0.001
15 minutes	AscSlope	4 (3.2 to 5.2)	4.1 (3.4 to 5)	0.676
	DescSlope	10.3 (9.6 to 11.3)	9.4 (8.3 to 10.2)	0.003
	NIRS VO_2_	153 (141 to 165)	141 (120 to 146)	0.005
30 minutes	AscSlope	4.2 (3.6 to 5.3)	3.4 (3.1 to 4.8)	0.006
	DescSlope	10.9 (9.5 to 12.6)	9.4 (7.4 to 10.4)	>0.001
	NIRS VO_2_	157 (122 to 171)	132 (112 to 152)	>0.001

**Table 2:** Effects of a repeat VOT on VOT-induced NIRS-derived variables in critically ill patients

Interval	Variable	First	Second	*P *value
5 minutes	AscSlope	3.6 (2.7 to 4)	3.4 (2.8 to 4.6)	0.065
	DescSlope	10 (8.4 to 11.6)	10.5 (8 to 11.8)	0.774
	NIRS VO_2_	103 (74 to 156)	108 (73 to 140)	0.442

## Conclusion

Rapid repetition of a VOT alters VOT-induced NIRS-derived variables in healthy volunteers but not in critically ill patients. If these alterations reflect preconditioning, our results suggest that this phenomenon may be altered in critically ill patients.

